# Fermented *Erigeron breviscapus* flavonoids: anti-pseudorabies virus efficacy and mechanisms *in vitro* and *in vivo*

**DOI:** 10.3389/fvets.2025.1562879

**Published:** 2025-04-16

**Authors:** Ying Zhang, Ting Li, Chunkun Yang, Qiong Pan, Changxu Pan, Xue Zhang, Ying Zhang, Xianghua Shu, Zheng Wang, Zhenghong He, Zichen Qu, Chunlian Song

**Affiliations:** ^1^College of Veterinary Medicine of Yunnan Agricultural University, Kunming, Yunnan, China; ^2^Yongshan County Animal Husbandry Technology Extension Station, Zhaotong, Yunnan, China; ^3^College of Animal Science and Technology of Yunnan Agricultural University, Kunming, Yunnan, China; ^4^Diqing Tibetan Autonomous Prefecture Animal Husbandry and Veterinary Scientific Research Institute, Shangri-la, Yunnan, China; ^5^Shangri-la Agriculture and Rural Bureau Animal Husbandry and Aquatic Technology Promotion Station, Shangri-la, Yunnan, China

**Keywords:** *Erigeron breviscapus*, fermented Chinese medicine, antiviral mode, PRV, viral brain inflammation

## Abstract

**Introduction:**

*Erigeron breviscapus* exhibits anti-inflammatory properties, protects neuronal cells and enhances immune function. Modern traditional Chinese medicine fermentation techniques can increase the bioactive compound content in *Erigeron breviscapus*. However, its potential therapeutic effects against the porcine pseudorabies virus (PRV) remain unclear.

**Methods:**

A PRV infection model was established in mouse trigeminal ganglion (TG) cells to determine the optimal antiviral mode of action of flavonoids from fermented *Erigeron breviscapus* (FEBF). Additionally, a PRV-infected rat model was developed to evaluate the *in vivo* antiviral efficacy of FEBF.

**Results:**

FEBF demonstrated a higher protective rate and a lower viral copy number compared to unfermented *E. breviscapus* flavonoids (EBF). The protective effect was most pronounced under toxicological and inhibitory conditions, surpassing the blocking effect. PRV infection upregulated TLR4, MyD88, and NF-κB p65 protein expression during the pre-infection phase, followed by their downregulation after 12 h. FEBF regulated PRV-induced changes in protein expression, restoring them to near-normal levels by 36 h. *In vivo* assessments of pathological injury, PRV viral load, neuronal count, and neuronal apoptosis indicated that FEBF provided superior neuroprotection compared to both Minocycline (MINO), a broad-spectrum neuroprotective drug, and unfermented *EBF.* Mechanistic studies further revealed that FEBF modulated microglial polarization and regulated the inflammatory cytokines IL-6, TNF-*α*, IL-4, and IL-10.

**Conclusion:**

These findings demonstrate that FEBF exhibits significant antiviral effects against PRV in both *in vitro* and *in vivo* models. FEBF represents a promising candidate for the development of anti-PRV therapeutics.

## 1 Introduction

Pseudorabies virus (PRV), also known as *α*-herpesvirus, is a linear, double-stranded DNA virus belonging to the *Herpesviridae* family (1). PRV infection in hosts results in microglial infiltration and necrosis (2), the formation of glial nodules around neurons, lysis of neuronal nuclei, aggregation of lymphocytes in vascular sections—forming the characteristic “vascular cuff phenomenon”—and the presence of acidic inclusion bodies in neuronal nuclei. Following recovery from PRV infection, pigs can develop lifelong latent infection in the trigeminal nerve (cranial nerve V), glossopharyngeal nerve (Cranial Nerve IX), and olfactory nerve (Cranial Nerve I) (3), without displaying clinical signs of latency (4).

*Erigeron breviscapus* (Vant.) Hand.-Mazz. (EB), an East Asian herbal medicine, has demonstrated a broad range of therapeutic effects, particularly in the treatment of central nervous system disorders (5). Flavonoids are the primary bioactive compounds in *Erigeron breviscapus* (6), and they exhibit neuroprotective, anti-inflammatory, anticancer, antioxidant, antibacterial, anti-inflammatory, and immunomodulatory functions (7). Studies have shown that breviscapine, a key flavonoid component, promotes the polarization of activated microglia from the M1 to M2 phenotype. Minocycline (MINO), a tetracycline antibiotic with high lipid solubility, is used as an inhibitor of microglial activation (8). It is known for its broad-spectrum antimicrobial activity and ability to cross the blood–brain barrier, where it exerts protective effects against neurological damage (9).

In this study, we investigated the inhibitory effects of fermented *Erigeron breviscapus* flavonoids (FEBF) and *Erigeron breviscapus* flavonoids (EBF) on PRV *in vitro* and *in vivo,* with the goal of identifying potential therapeutic agents for pseudorabies. Additionally, our findings provide a theoretical basis for the development of *Erigeron breviscapus* as an antiviral veterinary drug.

## 2 Materials and methods

### 2.1 Cells, viruses, and reagents

*Erigeron breviscapus* was obtained from Yaoan County, Chuxiong Prefecture, Yunnan Province, China. Whole plant material of *Erigeron breviscapus* was pulverized prior to extraction. The fermented breviscapine flavonoids were derived from the crude flavonoid liquid, which was sealed with paraffin wax and fermented at 36.59°C for 6 d. Flavonoid extraction from raw and fermented *Erigeron breviscapus* was performed using ultrasonic extraction with methanol, followed by rotary evaporation and freeze-drying to obtain flavonoid samples. The PRV-XD-F3 strain was donated by researcher Yao Jun from the Yunnan Academy of Animal Husbandry and Veterinary Science. Specific pathogen-free Sprague–Dawley (SD) rats (6–8 weeks old; equal numbers of males and females) were purchased from Kunming Medical University. The rats were kept in isolation for 7 d before the experiments.

### 2.2 Animal ethics and experimental design

This study investigated the *in vivo* and *in vitro* antiviral effects of FEBF in two parts.

(1) *In vitro* model: A PRV infection model was established by isolating mouse trigeminal ganglion progenitor cells

The experiment was allocated into four groups as detailed in Table 1.

- Blank control group (CTRL)
- PRV group (PRV)
- Fermented *Erigeron breviscapus* flavonoids group (FEBF)
- *Erigeron breviscapus* flavonoids group (EBF)

(2) *In vivo* model: A rat model of PRV infection was developed via intranasal inoculation of the virus.

**Table 1:** *In vitro* experimental design.

Group	Key steps (Pre-maintenance phase)	Cell collection (h post-maintenance)
Control	Maintenance medium only	0, 12, 24, 36, 48
PRV	100 × TCID₅₀ PRV (2 h) → Maintenance medium	0, 12, 24, 36, 48
FEF	4 mg/mL FEF (2 h) → Maintenance medium	0, 12, 24, 36, 48
PRV + FEF	100 × TCID₅₀ PRV (2 h) → PBS wash → 4 mg/mL FEF (2 h) → Maintenance medium	0, 12, 24, 36, 48

A total of 50 animals were randomized into five groups (*n* = 10 per group). The experimental design is summarized in Table 2.

- Blank control group (CTRL)
- PRV group (PRV)
- Minocycline group (MINO)
- *Erigeron breviscapus* flavonoids group (EBF)
- Fermented *Erigeron breviscapus* flavonoids group (FEBF)

**Table 2:** *In vivo* experimental design.

Group	Pre-treatment (Day 1–7)	Challenge (Day 7)	Post-challenge (Day 8–10)	Euthanasia
Control	Oral saline	PBS (40 μL, intranasal)	—	Day 10 (3 dpc)
PRV	Oral saline	PRV (40 μL, 1 × 10^7^·^32^ TCID₅₀/mL)	—	Day 10 (3 dpc)
EBF	1 g/kg/d oral	PRV (same as PRV group)	1 g/kg/d oral	Day 10 (3 dpc)
FEBF	1 g/kg/d oral	PRV (same as PRV group)	1 g/kg/d oral	Day 10 (3 dpc)
MINO	30 mg/kg/d oral	PRV (same as PRV group)	30 mg/kg/d oral	Day 10 (3 dpc)

We assessed three antiviral mechanisms:

(1) Blocking effect: Cells were pre-treated with different concentrations of the flavonoid solution for 2 h, after which the solution was removed. Cells were then inoculated with PRV and incubated for 2 h, washed with PBS and cultured in maintenance medium [The solution consists of DMEM supplemented with 1% fetal bovine serum (FBS)] for 48 h.
(2) Inhibitory effect: Cells were first inoculated with PRV and incubated for 2 h. After removal of the virus, cells were washed with PBS, treated with different concentrations of the flavonoid solution for 2 h, and then cultured in maintenance solution for 48 h.
(3) Direct killing: PRV was incubated with different concentrations of flavonoid solution for 2 h at 37°C before being added to cells. The mixture was incubated for 2 h, after which the cells were washed with PBS and further cultured in maintenance medium for 48 h.

The optimal antiviral strategy was determined by measuring the cell protection rate and viral load.

The experiment was divided into four groups: the blank control [CTRL, PRV group (PRV), the fermented *Erigeron breviscapus* flavonoid (FEBF) group, and the PRV + FEBF group]. The PRV and PRV + FEBF groups were inoculated with 100x TCID_50_ virus solution and incubated for 2 h. After incubation, the PRV group had its medium replaced with maintenance solution. The PRV + FEBF group was washed with PBS, followed by the addition of 4 mg/mL FEBF for 2 h before replacing the medium with maintenance solution. The FEBF group received 4 mg/mL FEBF for 2 h, after which the medium was replaced with maintenance solution. The time point of medium replacement was designated as 0 h, and cells were collected at 0, 12, 24, 36, and 48 h post-treatment. Protein expression levels of Toll-like receptor 4 (TLR4), myeloid differentiation primary response 88 (MyD88), and nuclear factor kappa B (NF-κB) p65 were analyzed (Table 3).

**Table 3:** Primers for PCRs used in this study.

Gene name	(5′ → 3′)	Product length (bP)	annealing temperature (°C)
β-actin	β-actin-F: CTGTGCTATGTTGCCCTAGACTTC	120	58.9
	β-actin-R: GAACCGCTCATTGCCGATAGTG	120	58.9
TNF-α	TNF-α-F:AGATGTGGAACTGGCAGAGGAG	131	58.7
	TNF-α-R:TCAGTAGACAGAAGAGCGTGGTG	131	58.7
IL-6	IL-6-F: CTTCCAGCCAGTTGCCTTCTTG	108	57.8
	IL-6-R:TGGTCTGTTGTGGGTGGTATCC	108	57.8
IL-4	IL-4-F:CAACAAGGAACACCACGGAGAAC	95	58.8
	IL-4-R:CTTCAAGCACGGAGGTACATCAC	95	58.8
IL-10	IL-10-F:GCAGTGGAGCAGGTGAAGAATG	104	57.7
	IL-10-R:ACGTAGGCTTCTATGCAGTTGATG	104	57.7
PRV-gE	gE121-qR CTACAGCGAGAGCGACAACGA	139	59.2
	gE121-qF CGACAGCGAGCAGATGACCA	139	59.2

All experiments performed in this study were approved by the International Animal Care and Use Committee of the Yunnan Agricultural University (Approval code: 202405003; approval date: January 1, 2022).

### 2.3 CCK-8 assay for cytoprotection rate

The cytosolic fraction from the first part of the experiment was collected, and the protection rate for each mode of action was calculated according to the manufacturer’s instructions for the CCK-8 kit.

### 2.4 RT-qPCR validation

The cytosolic fraction was collected, and PRV DNA was extracted from TG cells using a commercial DNA extraction kit. The viral copy number was analyzed using a SYBR Green-based absolute qPCR method with primers specific for the PRV gE gene (Table 1). The qPCR reaction conditions were as follows: pre-denaturation at 95°C for 5 min, followed by 40 cycles of denaturation at 95°C for 30 s, annealing at 59. 2°C for 30 s, and extension at 72°C for 30 s. A final extension was performed at 65°C for 5 s, and 96°C for 5 s. The standard curve for qPCR was established as Y = −3.679x + 35.72 (R^2^ = 0.999), where x represents the copy number and y denotes the Ct value.

Rat brain tissue from the second part of the experiment was homogenized in distilled water, followed by nucleic acid extraction (Novizen, China) and reverse transcription (Novizen, China) using a commercial kit. *β*-actin served as the internal control gene, and gene expression changes were analyzed using the 2^-ΔΔct^ method (refer to Table 3 for primers). All experiments were performed conducted following the manufacturer’s instructions.

### 2.5 Western blot assay

Total protein was extracted using RIPA lysis buffer containing 1% protease inhibitor. Protein concentrations were determined using the BAC method. Protein samples were mixed with loading buffer and denatured in a boiling water bath. Standard protein immunoblotting protocols were followed, utilizing primary antibodies against *β*-actin, MyD88, TRL4, and NF-κB p65 (rabbit-derived) at dilutions of 1:6000, 1:2000, 1:4000, and 1:3000, respectively. A sheep anti-rabbit secondary antibody was applied at a dilution of 1:5500. Target protein expression levels were quantified by calculating the ratio of the gray value of the target protein bands to that of the *β*-actin bands.

### 2.6 Elisa

Rat brain tissues from the second part of the experiment were homogenized in distilled water. The viral copy number and cytokine expression levels in the brain tissue were quantified using an ELISA kit, following the manufacturer’s instructions.

### 2.7 Brain tissue sectioning and H&E/Nissl/TUNEL staining

Brain tissue from Part II was fixed, routinely sectioned, and stained with hematoxylin and eosin (H&E). Nissl staining was performed using a toluidine blue solution (Solarbio, China), and a TUNEL staining assay was performed using a TUNEL staining kit (Solarbio, China) following the manufacturer’s instructions. Finally, a microscopic examination was performed.

### 2.8 Detection of microglial phenotypic polarization by indirect immunofluorescence methods

Brain tissues from Part II were fixed and sectioned, followed by the addition of primary antibodies (CD68 + Iba1, CD206 + Iba1, or Iba1 + NeuN). Fluorescent dyes TYR-520 (green) and TYR-570 (red) were diluted in TSA buffer at a ratio of 1:200. The stained sections were then observed and imaged using an inverted fluorescence microscope.

### 2.9 HPLC method

Chromatographic separation was performed using an Inert Sustain C18 column (4.6 × 150 mm, 5 μm). The mobile phase consisted of (A) 0.5% (v/v) formic acid aqueous solution and (B) acetonitrile, delivered at a flow rate of 0.8 mL/min. Detection was carried out at 335 nm. Calibration curves were established for lampblossom ethyl ether and its glycoside standards to quantify analytes. Subsequently, extracts of Lampsia officinalis flavonoids and fermented Lampsia officinalis flavonoids were analyzed by HPLC to determine the contents of lampsin and lampsin glycosides, respectively.

### 2.10 Statistical analyses

All data represent the results of at least three independent experiments and are expressed as mean ± standard deviation; GraphPad Prism 8.0 was used for statistical analysis of all data, and one-way ANOVA was used for analysis. 0.05 was considered statistically significant. Where appropriate, additional details are provided in the figure legends.

## 3 Results

### 3.1 Antiviral action of fermented *Erigeron breviscapus*

**Figure 1:**
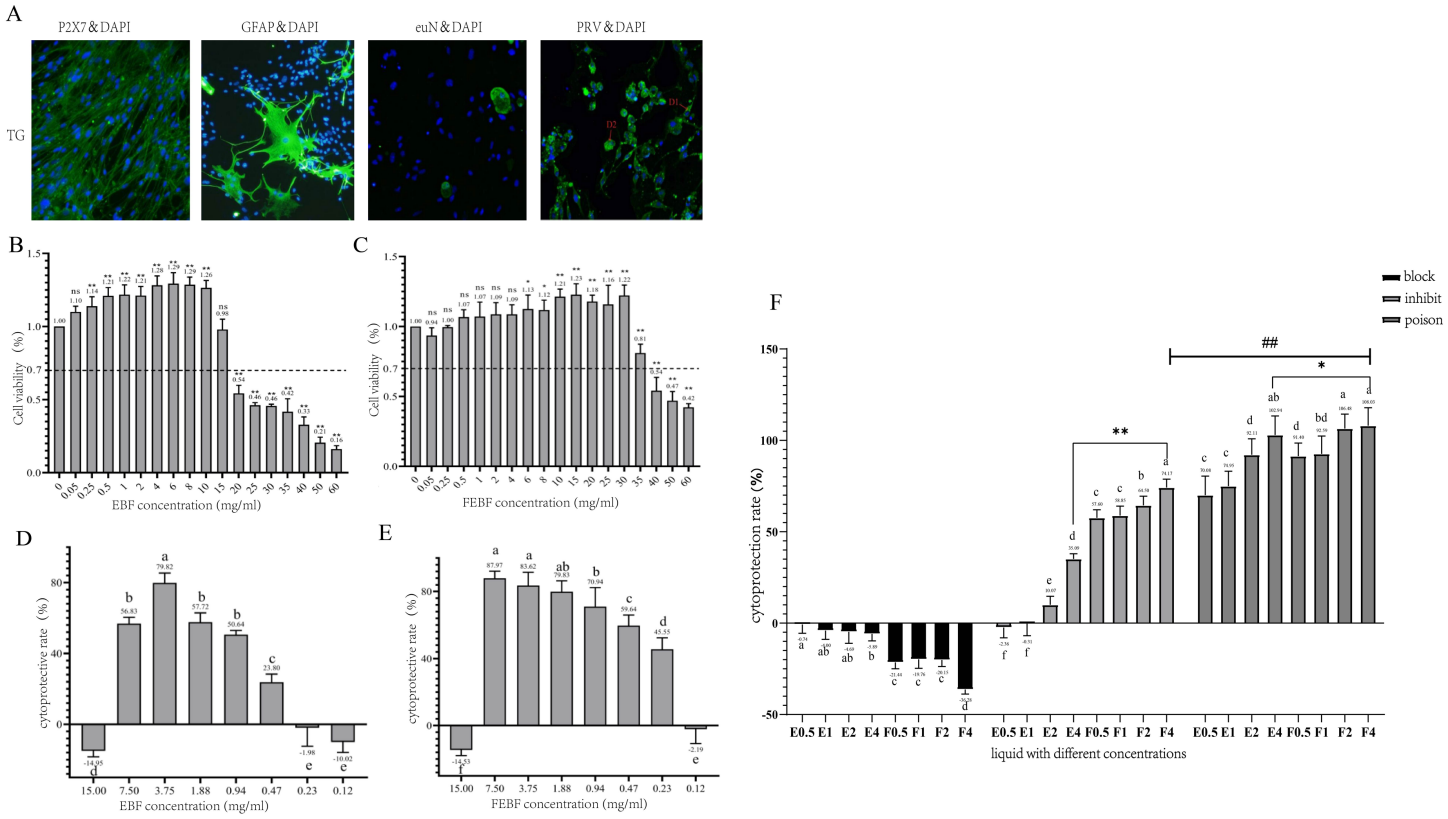
Antiviral effects of FEBF in three modalities. **(A)** Immunofluorescence results (100X). **(B)** Value added rate of cells by crude EBF. **(C)** Value added rate of cells by crude FEBF. **(D)** Protection rate of cells by crude EBF. **(E)** Protection rate of cells by crude FEBF. **(F)** Protection rate of cells by the drug in different modes and the effect of the drugs on the intracellular value added of PRV. **(B,C)**: ns indicates *p* > 0.05 vs. blank control; * indicates *p* < 0.05 vs. blank control; ** indicates *p* < 0.01 vs. blank control. **(D–G)**: a indicates *p* > 0.05; b indicates *p* < 0.05; c indicates *p* < 0.01. g: a indicates *p* > 0.05; b indicates *p* < 0.05; c indicates *p* < 0.01; * indicates E4 *p* < 0.05 vs F4; ** indicates E4 *p* < 0.01 vs F4; ### indicates blocking F4 *p* < 0.01 vs inhibiting F4.E1-4/F1-4:0.5, 1, 2, 4 mg/mL.

Trigeminal ganglion (TG) primary cell isolation and culture results were assessed using a GFAP immunofluorescence assay in mouse TG primary cells (Figure 1A). The cultured cells were identified as satellite glial cells, astrocytes, and other unidentified cells. The PRV positivity rate was 100%, with most viral particles located in the cytosol (D1) and a smaller proportion expressed in the nucleus (D2), suggesting that PRV could invade TG cells.

HPLC analysis revealed that the content of scutellarein aglycone reached 18 mg/g after fermentation treatment, representing a 13-fold increase compared to the EBF group. Toxicity studies demonstrated that prodrug concentrations ranging from 0.25 to 10 mg/mL and fermented flavonoid concentrations ranging from 6 to 30 mg/mL significantly increased the proliferation rate of TG cells The proliferation rate of TG cells exceeded 70% at concentrations ≤15 mg/mL in the prodrug group and ≤ 35 mg/mL in the fermentation group, confirming these as safe concentration thresholds. Therefore, the maximum safe concentrations for TG cells were determined to be 15 mg/mL in the prodrug group and 35 mg/mL in the fermentation group (Figures 1B,C).

Both non-fermented and fermented flavonoids from *Erigeron breviscapus* officinalis flowers inhibited PRV growth in a dose-dependent manner (Figures 1D–E). The minimum effective inhibitory concentrations of flavonoids from both the non-fermented and fermented *Erigeron breviscapus* officinalis flowers against PRV were 0.47 mg/mL and 0.23 mg/mL, respectively. The corresponding therapeutic indices (SI values) were calculated as follows:

- Original extract: SI = 15/0.47 = 31.91
- Fermented extract: SI = 35/0.23 = 152.17

These results suggest that both the original and fermented flavonoids are low-toxicity and effective antiviral agents. Notably, FEBF demonstrated superior antiviral efficacy compared to the original extract.

The results (Figure 1F) demonstrated that the protection rate of fermented *Erigeron breviscapus* was significantly or highly significantly higher than that of the original drug across all three modes of action: blocking, inhibition, and poisoning (*p* < 0.05 or *p* < 0.01). The protection rates for fermented *Erigeron breviscapus* were 91.40–108.42% under poisoning, 57.6–74.17% under inhibition, and negative under blocking. The protection rate under poisoning was significantly higher than that under inhibition (*p* < 0.01).

Regarding viral copy number, across the three modes of action—blocking, inhibiting and poisoning—the viral copy number in the fermented *Erigeron breviscapus* group was significantly lower than in the original *Erigeron breviscapus* group (*p* < 0.01). Furthermore, under poisoning, the viral copy number was significantly lower than under inhibition (*p* < 0.01). In all experiments, the optical density (OD) value increased with increasing concentration, while the viral copy number decreased correspondingly. The fermentation concentration of 4 mg/mL yielded the most effective anti-viral response.

### 3.2 Effect of fermented *Erigeron breviscapus* on PRV-activated TLR4, MyD88, NF-κB p65 signaling pathways

**Figure 2:**
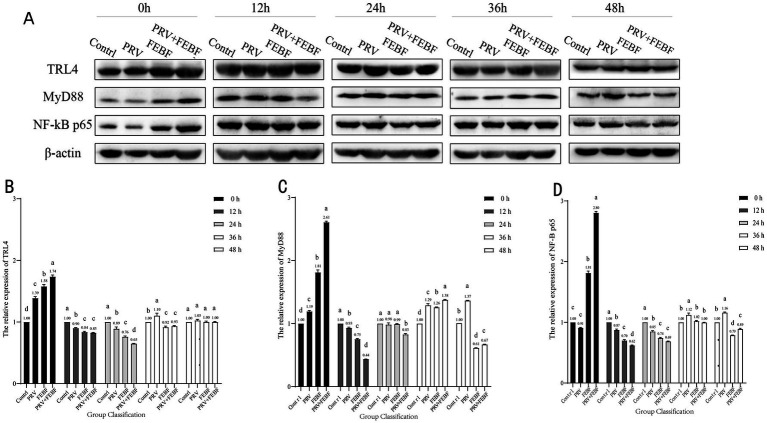
48 h dynamic changes of TG cell TLR4/MyD88/NF-κB p65 proteins. **(A)** Western Blot Analysis of Protein Expression. **(B)** 48 h dynamic changes of cellular TLR4 protein. **(C)** 48 h dynamic changes of cellular MyD88 protein. **(D)** 48 h dynamic changes of cellular NF-κB p65 protein. Comparison between groups a indicates *p* > 0.05; b indicates *p* < 0.05; c indicates *p* < 0.01.

Western blot (WB) analysis was used to detect the protein expression levels of TLR4, MyD88 and NF-κB p65 in infected cells, with the results presented in Figure 2. At 0 h post-infection (hpi), PRV infection caused a significant increase in the protein expression of TLR4, MyD88, and NF-κB p65 compared with the blank control group (*p* < 0.05). However, at 12 hpi and 24 hpi, PRV infection resulted in a significant downregulation of these proteins (*p* < 0.05). Following 36 h of fermentation, *Erigeron breviscapus* treatment, the regulation of TLR4, MyD88, NF-κB p65 protein expression was noted with their levels gradually returning to baseline.

### 3.3 Antiviral activity of *Erigeron breviscapus* flavonoids and fermented *Erigeron breviscapus* flavonoids in brain tissue of PRV-infected mice

**Figure 3:**
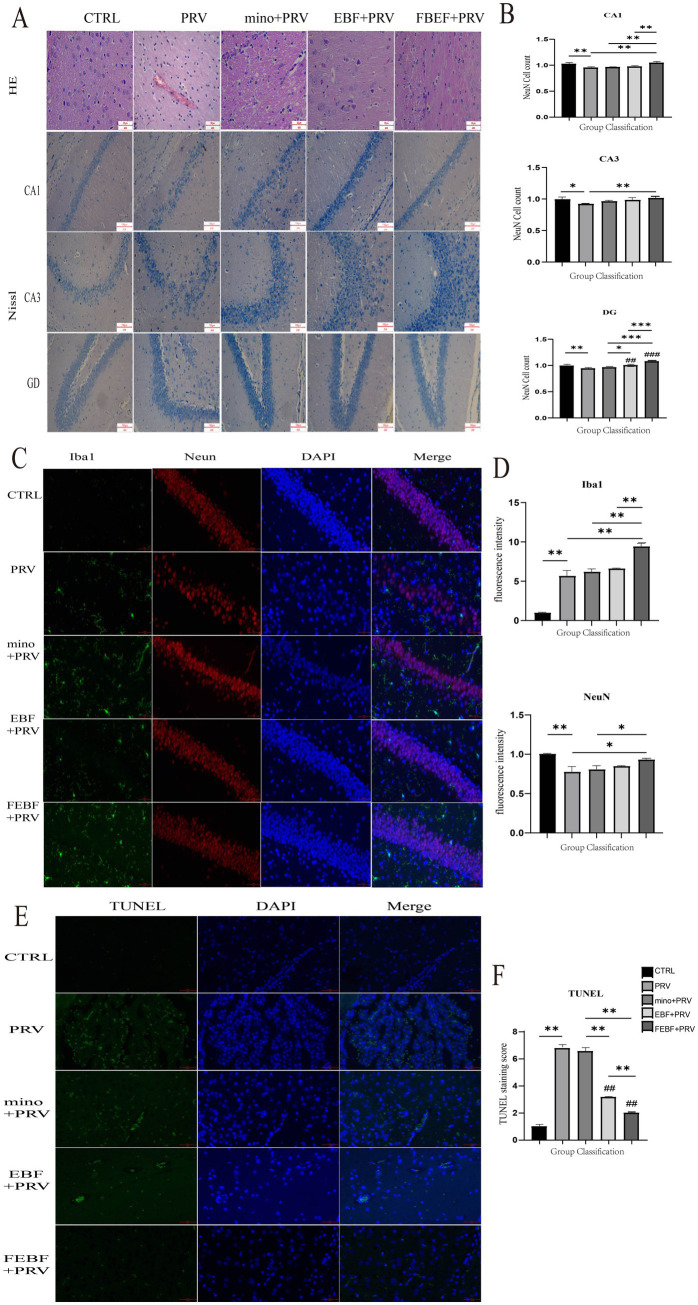
Antiviral activity of EBF and FEBF on brain tissues of PRV-infected mice. **(A)** Histopathological examination of rat brain tissue and Rat Brain Tissue CA1, CA3, DG Area Nichols Staining. **(B)** Number of Nitrosomes in CA1, CA3, and DG regions. **(C)** Effects of drugs on microglia and neurons in the CA1, CA3, and DG regions of the rat hippocampus, Co-staining of Iba1 and NeuN. F-G Relative fluorescence intensity was counted by using the ImageJ software. **(D)** The relative fluorescence intensities of Iba1 and NeuN were calculated using ImageJ software. **(E)** TUNEL staining of rat brain tissue. **(F)** Calculate the relative fluorescence intensity of TUNEL using ImageJ software. * indicates a significant difference (*p* < 0.05); ** indicates significant difference (*p* < 0.01).

Gross pathological changes in the rats were assessed based on clinical symptoms and pathological dissection (Figure 3). At 72 h post-infection, PRV-infected rats showed clinical symptoms, and three rats succumbed to the infection. Marked congestion and hemorrhage were observed in the brain tissues of infected rats. However, rats treated with any of the three drug formulations showed no overt clinical symptoms, and treatment with FEBF notably alleviated brain tissue congestion and hemorrhage.

HE staining was used to examine microscopic pathological changes in the brain tissue of PRV-infected rats (Figure 3A). The infected brain tissue exhibited perivascular lymphocyte infiltration, forming a characteristic cuff-like structure. Following treatment with fermented *Erigeron breviscapus*, neuronal morphology was restored to normal, and the scattered distribution of neuronal cells was preserved.

Nissl staining was performed to assess the morphology (Figure 3A) and neuronal cell density in the CA1, CA3 and dentate gyrus (DG) regions of rat hippocampus (Figure 3C). In the blank control group, neurons displayed normal morphology, were regular, densely packed, and contained abundant Nissl bodies in the cytoplasm. In PRV-infected rats, pyramidal cells were structurally disfigured, irregular in morphology, with enlarged and widened cell gap and haphazard arrangement. Treatment with fermented *Erigeron breviscapus* improved neuronal arrangement, reduced intercellular gaps, minimized the fracture zone, and restored structural integrity. Furthermore, FEBF provided superior neuroprotection in the CA1 and DG regions of the hippocampus compared to MINO.

The results are presented in Figures 3C,D. In the Iba1 and NeuN co-staining, PRV infection led to a highly significant increase in microglial fluorescence intensity compared to the blank group (*p* < 0.01). PRV-infected mice exhibited an increased number and size of microglia, as well as shorter and thicker processes. These changes corresponded with a highly significant decrease in neuronal fluorescence intensity (*p* < 0.01), a decrease in neuronal cell count, and an increase in intercellular gaps with unclear demarcation. Following treatment with fermented *Erigeron breviscapus*, neuronal fluorescence intensity significantly increased (*p* < 0.05). Additionally, the number of microglia and neurons significantly increased, microglial protrusions became elongated with an increased number of branches, and neuronal cells were more densely arranged with clear demarcation.

Terminal deoxynucleotidyl transferase dUTP nick-end labeling (TUNEL) staining was used to assess neuronal apoptosis in the cortical region of the brain, with results shown in Figures 3E,F. PRV infection significantly increased the number of apoptotic neurons in the PRV-infected group (*p* < 0.01). However, treatment with FEBF, EBF, and MINO significantly reduced neuronal apoptosis (*p* < 0.01). The reduction in apoptosis was particularly notable in groups treated with EBF and MINO (*p* < 0.01).

### 3.4 EBF and FEBF inhibit the inflammatory response following PRV infection

**Figure 4:**
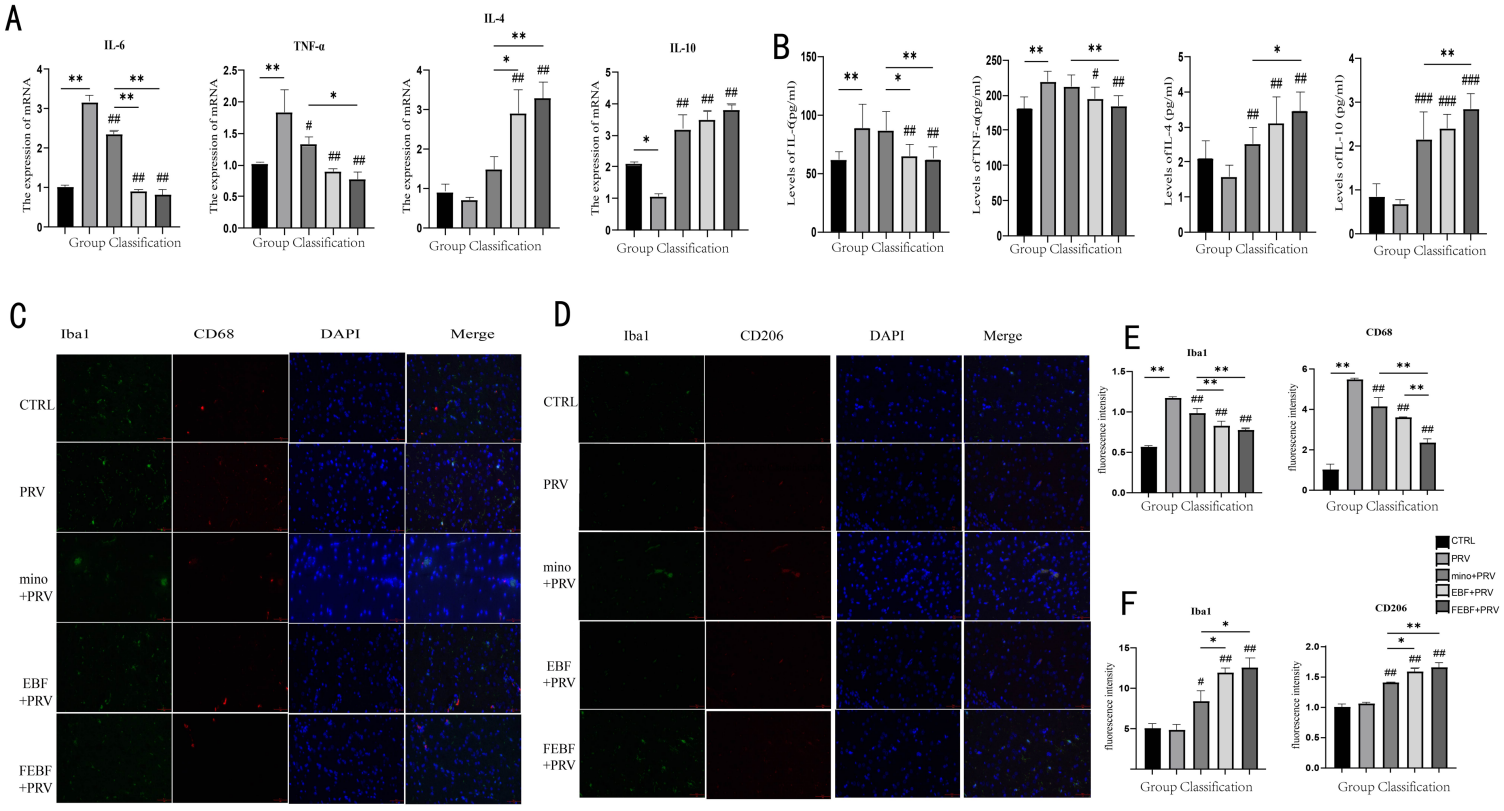
Drugs suppress the inflammatory response following PRV infection. **(A)** Inflammatory factor levels in brain tissue after drug administration. **(B)** Serum levels of inflammatory factors after drug administration. **(C)** Effects of drugs on the polarization of M1 microglia Co-staining of Iba1 and CD68. **(D)** Effects of drugs on the polarization of M2 microglia Co-staining of Iba1 and CD206. **(E)** Calculate the relative fluorescence intensity of Iba1 and CD68 using ImageJ software. **(F)** Calculate the relative fluorescence intensity of Iba1 and CD206 using ImageJ software. **Indicates significant difference (*p* < 0.01); ## indicates (*p* < 0.01) vs. PRV group.

The expression levels of inflammatory factors in rat brain tissue were measured using qPCR (Figure 4A), while their levels in rat serum were assessed using ELISA (Figure 4B). As shown in the figures, PRV infection led to a marked increase in the gene expression levels of pro-inflammatory cytokines IL-6 and TNF-*α* in both brain tissue and serum. Conversely, the levels of anti-inflammatory cytokines IL-4 and IL-10 were significantly decreased following PRV infection. Treatment with FEBF, EBF, and MINO significantly or highly significantly upregulated the expression of IL-4 and IL-10 in both brain tissue and serum (*p* < 0.05 or *p* < 0.01). Additionally, these treatments led to a highly significant downregulation of IL-6 and TNF-α in both compartments (*p* < 0.01). Notably, FEBF exhibited superior efficacy in modulating inflammatory cytokines compared to EBF and MINO.

Indirect immunofluorescence staining was used to observe microglial polarization, with results presented in Figures 4C–F. In Iba1 and CD68 co-staining, PRV infection significantly increased the fluorescence intensity of both total microglia and M1-type microglia (*p* < 0.01). However, following treatment with MINO, EBF, and FEBF, the fluorescence intensity of microglia and M1-type cells was significantly reduced (*p* < 0.01). Among the treatment groups, FEBF most effectively attenuated the fluorescence intensity of microglia and M1-type cells compared to EBF and MINO (*p* < 0.01).

The fluorescence intensity of M2-type microglia was significantly or highly significantly increased following PRV infection (*p* < 0.05, *p* < 0.01, respectively). Treatment with MINO, EBF, and FEBF further increased the fluorescence intensity of M2-type microglia (*p* < 0.05, *p* < 0.01). Notably, the FEBF group exhibited a greater enhancement in M1-type microglial fluorescence intensity compared to the EBF and MINO groups (*p* > 0.01).

Please use the corresponding catalog number and RRID in the current manuscript to participate in the Resource Identification Initiative. For more information about the project and the steps for searching for an RRID, please click http://www.frontiersin.org/files/pdf/letter_to_author.pdf.

## 4 Discussion

PRV is a typical neurophilic virus (10), and Prior experimental studies have demonstrated that it predominantly localizes in neurons, microglia, nerve fibers, the cerebellar granule layer, and the Purkinje cell layer (3). PRV invades the central nervous system through a pathway that extends from the cerebrum to the cerebellum and, ultimately, to the brainstem, with the severity of brain damage increasing over time. The viral infection progresses from the olfactory bulb to the hippocampus and, ultimately to the medulla oblongata, with viral expression rising over time (3). In this study, we investigated the anti-PRV effects of fermented *Erigeron breviscapus* on TG cells *in vitro*, focusing mainly on toxic and inhibitory phases. Our findings indicate that fermented *Erigeron breviscapus* modulates the TLR4/MyD88/NF-κB signaling pathway, which is altered by PRV infection in TG cells. Moreover, *in vivo* experiments demonstrated that fermented *Erigeron breviscapus* plays a protective role in PRV-infected mouse brain tissue, mitigating pathological damage, reducing viral load, and shifting microglial polarization from a pro-inflammatory to an anti-inflammatory state.

Following acute infection, PRV establishes lifelong latent infection in the porcine trigeminal ganglion, making the development of PRV-infected TG progenitor models particularly crucial (11). In this study, we identified TG progenitor cells as comprising 65% satellite glial cells, 3% astrocytes, 8% neurons, and 24% unidentified cells based on specific markers and morphological characterization. We found that P2X7 was stably expressed in satellite glial cells. These results align with those of Belzer et al. (26), who mapped mouse trigeminal ganglion cells through genomics and identified eight neuronal and seven non-neuronal subtypes. The findings of this study are consistent with previous studies, confirming the reliability of our TG cell culture method. Our cell characterization analysis further demonstrated that the cell composition in trigeminal ganglion precursors closely resembles that of cerebral tissue, reinforcing the notion that PRV latency in the trigeminal ganglion is facilitated by this high degree of cellular similarity. This cellular consistency may also contribute to the ability of PRV to spread along the trigeminal ganglion. Consequently, using ganglion progenitor cells as an *in vitro* model is vital for studying PRV pathogenesis and latency mechanisms.

Fermented traditional Chinese medicine offers several advantages, including safety, high efficiency, low toxicity, and no residual effects, making it an excellent alternative to antibiotics and a potent growth-promoting agent. It plays a crucial role in promoting the green, sustainable, and healthy development of animal husbandry (12). Fermented hawthorn extract has been shown to significantly reduce body weight, alleviate high-fat diet-induced nonalcoholic fatty liver disease in rats, and show significant antioxidant effects (13). Furthermore, this study found that the antiviral efficacy of FEBF was significantly superior to that of non-FEBF in both *in vivo* and *in vitro* models. This suggests that the fermentation process enhances the therapeutic potential of EBF.

Flavonoids are among the active components of numerous traditional Chinese and natural medicines, exhibiting a wide range of biological activities, including anti-inflammatory, antibacterial, antiviral, hypoglycemic, hypolipidemic, antioxidant, and immunomodulatory effects (14). Increasing research has been conducted on flavonoids as potential antiviral agents against PRV (15). Li et al. (16) reported that kaempferol inhibits PRV replication *in vitro* in a dose-dependent manner while also enhancing the survival of PRV-infected mice *in vivo*. Breviscapine, a flavonoid compound, has been found to improve apoptosis levels and enhance the expression of synaptic markers in rats with traumatic brain injury (17). However, the antiviral properties of EBF have been largely understudied.

The viral replication process typically involves several stages, including adsorption, penetration, replication, and release. Inhibiting any of these steps can effectively suppress viral infection. Our findings demonstrate that lampbloom and FEBF significantly inhibit PRV replication and show virucidal activity in a dose-dependent manner, suggesting that fermentation enhances the antiviral activity of EBF. Interestingly, qPCR analysis in the blocking mode revealed that the OD values in both treatment groups were significantly lower than those in the blank control group, with OD values decreasing as drug concentration increased. This finding suggests that pretreatment with calendula and FEBF exacerbates PRV-induced cellular damage. Further studies are warranted to explore the optimal dosing regimen and to elucidate the mechanism by which this blocking mode intensifies cellular lesions.

The TLR4 signaling pathway consists of two branches: the MyD88-dependent pathway and the MyD88-independent pathway, both of which can induce NF-κB phosphorylation. Notably, NF-κB p65 serves as a marker for NF-κB phosphorylation (18). Ultimately, this pathway leads to the production of numerous cytokines and inflammatory factors, which play crucial roles in immune responses, inflammatory processes, and anti-apoptotic mechanisms (19). Previous studies have demonstrated that the NF-κB signaling pathway is activated in the trigeminal ganglion of PRV-infected mice. This activation occurs in a biphasic manner—initially through downregulation, followed by the upregulation of NF-κB p65, MyD88 and the TIR-domain-containing adaptor-inducing interferon-*β* (TRIF). Additionally, the pathway promotes phosphorylation of I κBɑ and NF-κB p65 (18). In contrast to the PRV-infected group in this study, the TLR4/MyD88/NF-κB signaling pathway exhibited significant upregulation at 0 h, followed by a downward trend before rising again. Cheng et al. investigated the expression levels of TLR4/MyD88/NF-κB in the brain and CYP3A4 in the intestinal segments of rats. Their findings revealed that lamprophyllin could protect against cerebral ischemia–reperfusion injury by regulating intestinal microbiota, inhibiting TLR4/MyD88/NF-κB inflammatory pathways in the brain, and regulating intestinal CYP3A4 expression (20).

In this study, we investigated the dynamic changes in TLR4, MyD88, and NF-κB p65 expression in PRV-infected TG cells treated with EBF and FEBF. Our results indicate that FEBF effectively downregulated TLR4, MyD88, and NF-κB p65 protein expression during the early phase of infection. Furthermore, they mitigated PRV-induced upregulation of these proteins in the later stages of the disease. Notably, the antiviral and regulatory effects of FEBF were superior to those of non-FEBF.

Studies have shown that PRV primarily infects neuronal cells and can induce severe neuroinflammation in the brain (21). Recent findings on PRV-induced neuroinflammatory responses in mice suggest that modulating inflammation is a crucial mechanism in combatting PRV infection (22). Since M1 microglia are typically pro-inflammatory, whereas M2 microglia are anti-inflammatory and neuroprotective, shifting the balance toward M2 microglia can help mitigate local inflammatory responses. Notably, breviscapine has been shown to promote M2 polarization by inhibiting the JNK and P38 pathways and simultaneously enhancing the ERK1/2 pathway (23). Microglia play a key role in regulating the balance between inflammatory activation and suppression (24). The polarization of Microglia into M1 or M2 phenotypes is characterized by the expression of specific markers: M1 (CD68) and M2 (CD206). Therefore, modulating microglial polarization is an essential strategy for controlling neuroinflammation (25). The findings of this study demonstrate that FEBF effectively regulates microglial polarization by shifting them from M1 to M2. This shift results in the inhibition of CD68 expression and the upregulation of CD206 expression, thereby enhancing the anti-inflammatory and neuroprotective functions of M2 microglia. Consequently, this modulation helps alleviate PRV-induced neuroinflammation in brain tissue. Moreover, FEBF exhibited superior anti-inflammatory effects compared to non-FEBF.

While this study demonstrates the enhanced antiviral efficacy of fermented *Erigeron breviscapus* flavone compared to its non-fermented form, we acknowledge that the absence of direct comparison with currently approved anti-PRV drugs (e.g., acyclovir or ganciclovir) limits the clinical relevance of our findings. Future studies will focus on evaluating the relative potency, safety profiles, and cost-effectiveness of fermented *Erigeron breviscapus* flavone against established antiviral therapies to comprehensively assess its therapeutic potential.

## 5 Conclusion

This study demonstrated that FEBF exhibit significant anti-PRV activity *in vitro*, surpassing the antiviral efficacy of non-FEBF. The antiviral effects were mainly achieved through the inhibition of viral replication and direct virulence in a dose-dependent manner. Furthermore, FEBF effectively suppressed the PRV-induced upregulation of TLR4, MyD88, and NF-κB p65 proteins between 36 and 48 h post-infection. *In vivo*, these flavonoids inhibited PRV proliferation in the mouse brain and mitigated infection-induced inflammatory responses. These findings suggest that FEBF may offer a promising therapeutic strategy for managing PRV infections.

## Data Availability

The original contributions presented in the study are included in the article/supplementary material, further inquiries can be directed to the corresponding author/s.
